# Switching from allopurinol to febuxostat for the treatment of hyperuricemia and renal function in patients with chronic kidney disease

**DOI:** 10.1007/s10067-014-2745-5

**Published:** 2014-07-22

**Authors:** Yuki Tsuruta, Toshio Mochizuki, Takahito Moriyama, Mitsuyo Itabashi, Takashi Takei, Ken Tsuchiya, Kosaku Nitta

**Affiliations:** Department of Medicine, Kidney Center, Tokyo Women’s Medical University, 8-1 Kawada-cho, Shinjuku-ku, Tokyo, 162-8666 Japan

**Keywords:** Allopurinol, Chronic kidney disease, eGFR, Febuxostat, Hyperuricemia, Uric acid

## Abstract

Hyperuricemia is a frequent complication of chronic kidney disease (CKD). Febuxostat is a novel xanthine oxidase inhibitor that is metabolized by many metabolic pathways in the kidney and the liver. We performed a 1-year cohort study of 73 hyperuricemic patients who had an estimated glomerular filtration rate (eGFR) below 45 ml/min and were being treated with urate-lowering therapy. In 51 patients, treatment was changed from allopurinol to febuxostat, and the other 22 patients were continued on allopurinol. The serum levels of uric acid (UA) level, creatinine, and other biochemical parameters were measured at baseline and after 3, 6, 9, and 12 months of treatment. The serum UA levels significantly decreased from 6.1 ± 1.0 to 5.7 ± 1.2 mg/dl in the febuxostat group and significantly increased from 6.2 ± 1.1 to 6.6 ± 1.1 mg/dl in the allopurinol group. The eGFR decreased 27.3 to 25.7 ml/min in the febuxostat group and from 26.1 to 19.9 ml/min in the allopurinol group. The switch from allopurinol to febuxostat was significantly associated with the changes in eGFR according to a multiple regression analysis (*β* = −0.22145, *P* < 0.05). Febuxostat reduced the serum UA levels and slowed the progression of renal disease in our CKD cohort in comparison with allopurinol.

## Introduction

Hyperuricemia is a frequent complication of chronic kidney disease (CKD) and with other risk factors for CKD, such as hypertension and metabolic syndrome [1]. Uric acid (UA) is the end product of purine metabolism in humans, and approximately 70 % of UA is eliminated by urinary excretion. Because urinary UA excretion is decreased in CKD patients, the prevalence of hyperuricemia is higher [2]. However, hyperuricemia has not been considered to have a causal role in CKD [3]. Evidence that hyperuricemia is an independent risk factor for CKD has been increasing in the past few years [4]. In an epidemiologic study of a healthy population of 21,475 subjects who were followed for 7 years, hyperuricemia was found to independently increase the risk of new onset CKD [5].

CKD is associated with strongly and consistently with cardiovascular disease (CVD) and mortality [6]. The mechanism through which UA is regulated by the kidney and the relationship between UA, kidney function, and CVD are not fully understood [7]. Recently, evidence has accumulated showing that hyperuricemia has a role in the pathogenesis of CVD and the progression of CKD, indicating the necessity for treatment even in the absence of symptoms of gouty arthritis [8, 9]. However, a major challenge in treating patients with hyperuricemia is the occurrence of drug-related adverse effects that are often augmented in the presence of kidney dysfunction [10].

Pharmacologic options for urate-lowering therapy consist of urate synthesis inhibitors and uricosuric agents. Most uricosuric agents are not indicated for patients with CKD because of their mechanism of action; hyperuricemia in CKD patients is mainly treated with xanthine oxidase (XO) inhibitors [11]. Allopurinol is metabolized by aldehyde oxidase to oxypurinol, which is also a XO inhibitor. Oxypurinol is then excreted via the kidney; its efficacy is insufficient, and the risk of adverse events is higher in some CKD patients [12]. Febuxostat is a novel XO inhibitor that became available clinically in 2011 in Japan, and it is metabolized by many metabolic pathways in the kidney and the liver.

The aim of this study was to evaluate the effects of switching the treatment of hyperuricemic patients with CKD from allopurinol to febuxostat on the serum UA levels and the progression of their renal disease.

## Methods

### Patients

We performed a 1-year retrospective observational study of 73 hyperuricemic patients who had an estimated glomerular filtration rate (eGFR) below 45 ml/min and were being treated with urate-lowering therapy. The criteria for inclusion as subjects of this study were the following: (1) presence of CKD as manifested by an eGFR below 45 ml/min, (2) current urate-lowering therapy with allopurinol, (3) stable renal function (no increase in baseline serum creatinine by 50 % in the previous 3 months), and (4) stable clinical condition (no hospitalization in the previous 3 months).

A total of 85 patients in our renal clinic had an eGFR <45 ml/min and were being treated with allopurinol at the time of entry. One patient had been hospitalized within 3 months before start of this study. Febuxostat was prescribed for 57 patients of the remaining 84 patients, and they constituted the febuxostat group. The other 27 patients continued to be treated with allopurinol and constituted the allopurinol group. Three nephrologists decided on the switch from allopurinol to febuxostat and the dose of prescription in consideration of serum UA levels, renal function, and requirements of the patients. All of the subjects in the febuxostat group began the treatment with febuxostat between April 2012 and December 2012.

The baseline demographic data, laboratory data, and information concerning comorbid conditions and medication were collected at the time of entry, and serum levels of UA and creatinine were measured 3, 6, 9, and 12 months after the start of the study. The eGFR was calculated using a formula based on serum creatinine (Cr) developed by the Japanese Society of Nephrology for the Japanese population [eGFR (ml/min/1.73 m^2^) = 194 × Cr^−1.094^ × age^−0.287^ (×0.739 if women) [13]. The study was performed in accordance with the Declaration of Helsinki and the Principles of Good Clinical Practice. The Institutional Research Ethics Committee approved the study protocol (No. 1841).

### Endpoints

The primary endpoint of interest was serum UA levels after 1 year from the switch from allopurinol to febuxostat. The secondary endpoint was the changes in eGFR in the febuxostat group and allopurinol group.

### Statistical analysis

Continuous variables are reported as the mean ± SD, and categorical variables are reported as percentages unless otherwise stated. Non-paired two-tailed Student’s *t* tests were used to compare continuous variables between the febuxostat group and allopurinol group. Paired two-tailed Student’s *t* tests were used to compare pre-treatment and post-treatment values. Multiple regression analysis was performed by making eGFR the dependent variable to estimate the effect of the switch from allopurinol to febuxostat on eGFR. *P* values less than 0.05 were considered to indicate statistical significance. All analyses were performed with the JMP for Windows software program (SAS Institute, Cary, NC, USA).

## Results

Eighty-four patients were enrolled in the study. This was a retrospective observational cohort study. Treatment of 57 patients was switched from allopurinol to febuxostat, and the other 27 patients were continued on allopurinol. During the 1-year observation period, five patients (8.8 %) in the febuxostat group and four patients (14.8 %) in the allopurinol group developed indications for hemodialysis. One patient in the febuxostat group was transferred to a different renal clinic, and one patient in the allopurinol group died a sudden death; both patients were excluded from the study for comparison of the changes in renal function.

The baseline characteristics, including laboratory data, of the both groups are shown in Table 1. There were no significant differences in clinical parameters in the febuxostat group and allopurinol group, but the mean age of the patients in the febuxostat group tended to be lower, and they had lower percentages of nephrosclerosis and diabetic nephropathy as an etiology of CKD.

**Table 1:** Baseline characteristics of patients in the febuxostat group and allopurinol group

	Febuxostat group (*n* = 51)	Allopurinol group (*n* = 22)
Age (years)	67.4 ± 12.3	72.9 ± 10.7
Gender (M:F)	29:22	16:6
Body weight (kg)	58.0 ± 13.3	59.3 ± 9.8
Hemoglobin (g/dl)	11.9 ± 1.8	11.5 ± 1.9
Serum albumin (g/dl)	4.0 ± 0.5	3.8 ± 0.4
Serum creatinine (mg/dl)	2.2 ± 1.1	2.1 ± 0.7
eGFR (ml/min per 1.73 m^2^)	27.2 ± 10.5	26.2 ± 9.2
Uric acid (mg/dl)	6.1 ± 0.9	6.2 ± 1.1
Etiology of renal disease (%)		
Diabetes mellitus	6	18
Nephrosclerosis	29	55
Glomerulonephritis	35	14
Others	30	13
RAAS blockers (%)	77	86

Variables are presented as mean ± SD

No significant differences were observed between the two groups

*RAAS* renin-angiotensin-aldosterone system

### Changes in serum UA level of each drug group

The changes in serum UA levels in each group between 3 months before and 12 months after the time of entry are shown in Table 2. The difference between the serum UA levels in the two groups before the start of the study was not significant. The serum UA levels had significantly decreased after 9 months in the febuxostat group but had significantly increased after 12 months in the allopurinol group, and as a result, the serum UA level in the febuxostat group was significantly lower than in the allopurinol group at 9 months after switching the drug. There was no significant difference in the rate of achievement of the target serum UA level (<6.0 mg/dl) between the two groups at the start of this study (45.1 % in the febuxostat group vs 50 % in the allopurinol group, *P* = 0.70), but after 1-year of treatment, in this study, the achievement rate was significantly higher in the febuxostat group than in the allopurinol group (68.6 % vs 31.8 %, *P* < 0.01).

**Table 2:** Effect of the switch from allopurinol to febuxostat on serum UA levels

	Febuxostatgroup (*n* = 51)	Allopurinol group (*n* = 22)	*P* value**
Before 3 months	6.2 ± 0.9	6.4 ± 0.7	NS
Basal	6.1 ± 1.0	6.2 ± 1.1	NS
3 months	5.9 ± 1.3	6.2 ± 0.7	NS
6 months	5.8 ± 1.2	6.3 ± 1.0	NS
9 months	5.7 ± 1.3*	6.4 ± 1.0	<0.05
12 months	5.7 ± 1.2*	6.6 ± 1.1*	<0.01

Variables are presented as mean ± SD

*significant differences (*P* < 0.05) in comparison to baseline period wtihin each group

**difference in comparison to each group within same period

### Changes in serum UA level of each drug dosage

**Fig. 1:**
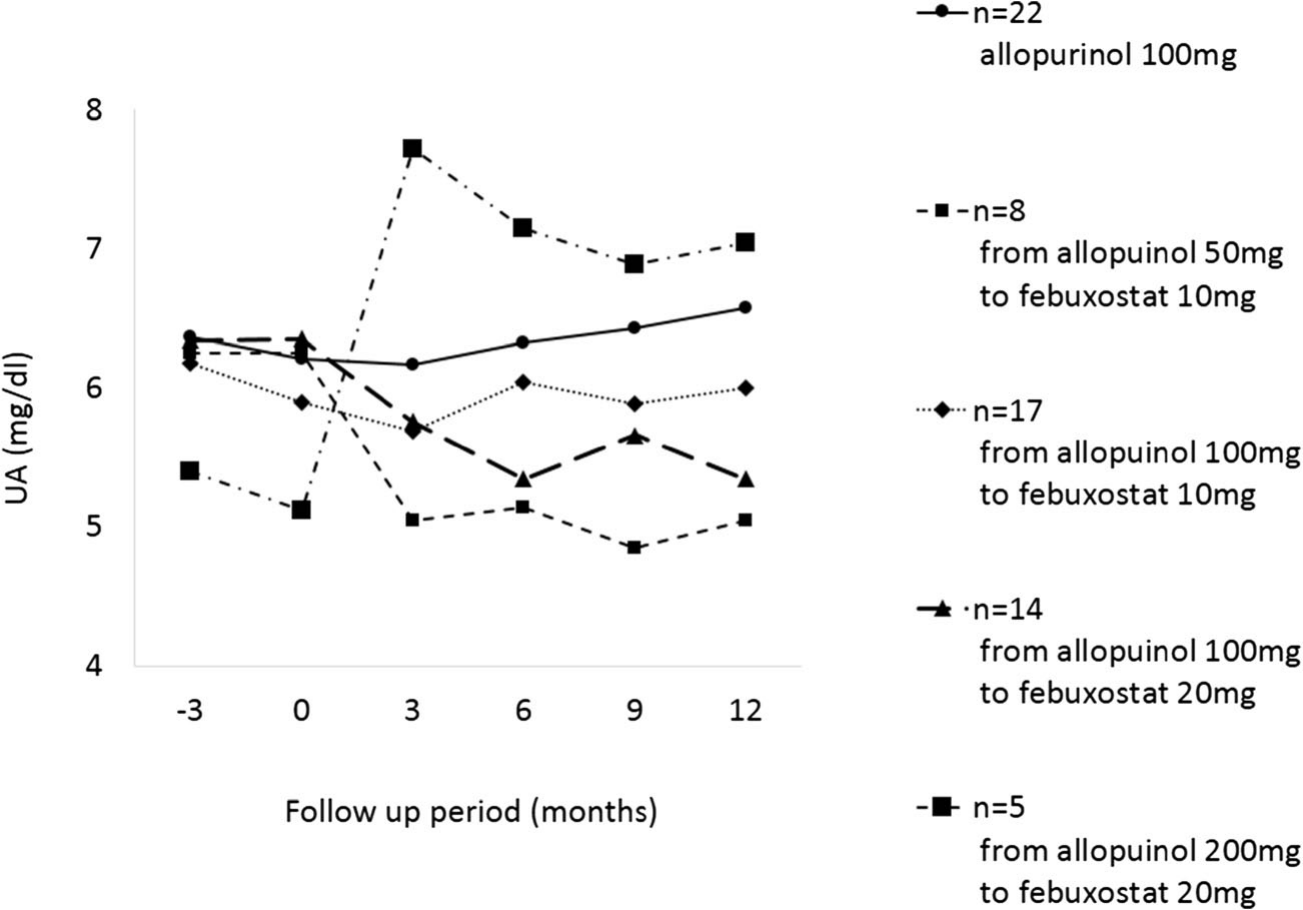
Changes in serum UA levels in the each drug dosage group. Values are expressed as mean values

Figure 1 shows the changes in serum UA levels during the observation period in the patient group. Two patients whose dose had changed after switching to febuxostat, one patient who had been switched from allopurinol 200 mg/day to febuxostat 10 mg/day, one patient who had been switched from allopurinol 200 mg/day to febuxostat 40 mg/day, and three patients who were switched to combination therapy were excluded in this figure. These results show that the 1000-mg dose of allopurinol and the 10-mg dose of febuxostat have essentially equivalent effect. On the other hand, the switch from allopurinol 200 mg/day to febuxostat 20 mg/day resulted in an increase serum UA level.

### Progression of renal disease

The changes in eGFR in the two groups between 3 months before and 12 months after the time of entry are shown in Table 3. There was no significant difference in the eGFR values of the two groups before 3 months and at the start of this study. After 9 months, the eGFR had decreased in both groups but had decreased more in the allopurinol group, and as a result, the eGFR was significantly higher in the febuxostat group than in the allopurinol group after 1-year treatment. In the febuxostat group, 12 (23 %) patients increased eGFR, but in the allopurinol group, there was no patient that increased eGFR. The results of a multiple regression analysis that included age, gender, hemoglobin, albumin, eGFR, prevalence of diabetes, use of renin-angiotensin-aldosterone blockers, and the switch from allopurinol to febuxostat showed that treatment with febuxostat independently associated with the change in eGFR (*β* = 0.22145, *P* < 0.05, Table. 4).

**Table 3:** Effect of the switch from allopurinol to febuxostat on changes in eGFR

	Febuxostat group (*n* = 51)	Allopurinol group (*n* = 22)	*P* value**
Before 3 months	29.3 ± 11.5	25.9 ± 8.9	NS
Basal	27.3 ± 10.6	26.1 ± 9.2	NS
3 months	26.4 ± 10.5	24.2 ± 9.4	NS
6 months	26.0 ± 10.4*	23.3 ± 9.4*	NS
9 months	25.9 ± 11.0*	22.0 ± 9.2*	NS
12 months	25.7 ± 11.3*	19.9 ± 9.5*	<0.05

Variables are presented as mean ± SD

*significant differences (*P* < 0.05) in comparison with baseline period within each group

**difference in comparison to each group within same period

**Table 4:** Multiple regression analysis for detecting independent variables associated with the changes in eGFR

	*β* value	*P* value
Age (years)	−0.06799	0.4952
Gender (M)	0.13059	0.2027
Hemoglobin (g/dl)	0.079493	0.5471
Serum albumin (g/dl)	−0.04083	0.717
eGFR (ml/min per 1.73 m^2^)	0.453673	0.0004
Diabetes nephropathy	−0.02244	0.8286
RAAS blockers (%)	0.154734	0.1261
Switch to febuxostat	0.22145	0.0375

R2 = 0.4245 in this model

*RAAS* renin-angiotensin-aldosterone system

### Adverse events

Liver function abnormality (elevation of transaminase) was observed in one patient of febuxostat group, and a mild attack of gout was observed in one patient during 3 months after the time of entry in the febuxostat group. However, both adverse events were soon resolved, and treatment was not discontinued because of improvement in both groups.

## Discussion

Hyperuricemia is a frequent complication of CKD but also is a risk factor for CVD, and CKD is considered a very important risk factor for CVD [7]. Consequently, it is very difficult to independently evaluate the effect of hyperuricemia on the progression of CKD, the risk of CVD, and mortality [14–16]. Recent epidemiologic studies indicated that UA had a causal role in the progression of renal disease in hypertensive women [17], a normotensive population [18], and diabetic patients [19]. A large epidemiologic study of 177,570 individuals for a total of 527,597 person-years revealed that a higher serum UA level was an independent risk factor for end-stage renal disease (HR 2.14 [1.65–2.77] for highest vs lowest quartile) [20].

Several mechanisms have been postulated for the causal role of UA in the progression of renal disease. One of the postulated mechanism is that hyperuricemia induces endothelial dysfunction because of a reduction in nitric oxide. The serum nitric oxide level decreases when hyperuricemia is induced in rats, and the decrease is reversed after administration of allopurinol [21]. Another postulated mechanism is that hyperuricemia stimulates the renin-angiotensin system and proliferation of vascular smooth muscle cells (VSMCs) [22]. Incubation of rat VSMCs with UA stimulated the proliferation of VSMC and increased anigiotensinogen messenger RNA expression, and both responses were prevented by losartan and captopril.

Several studies in the past few years have evaluated the effect of urate-lowering therapy on renal function. A randomized control trial conducted on 113 patients with an eGFR <60 ml/min showed that allopurinol decreased the serum UA level and had a renoprotective effect [23]. After 24 months in the control group, the eGFR had decreased from 39.5 to 35.9 ml/min but increased from 40.8 to 42.2 ml/min in the allopurinol group. The serum C-reactive protein level and prevalence of CVD decreased in the allopurinol group. Siu et al. also reported observing a renoprotective effect of allopurinol after 1 year of treatment in a randomized control trial conducted on 54 patients with a serum creatinine level above 1.35 mg/d1 [24].

Allopurinol has long been regarded as the only XO inhibitor drug, but allopurinol is metabolized in the kidney; the dosage must be reduced in patients with CKD. By contrast, since febuxostat is metabolized by more than one metabolic pathway in the kidney and liver, it is not necessary to reduce the dosage in patents with CKD [25]. Sakai et al. have recently reported that febuxostat is effective for treating allopurinol-resistant hyperuricemia in CKD patients [26]. Whereas the eGFR slope was negative during allopurinol treatment, it became positive after the switch to febuxostat. The efficacy and tolerability of the switch from allopurinol to febuxostat in patients with moderate CKD in this study have demonstrated useful information for clinical practice in renal clinic. On the other hand, Stamp et al. showed the efficacy and safety of increasing the allopurinol dose above the recommendation dose of creatinine clearance-based dosing guidelines in CKD patients with a mean creatinine clearance 62.2 ml/min [27]. Therefore, it is important to compare the renoprotective effects of allopurinol and febuxostat in a large cohort of CKD patients.

There were several limitations to the present study. The first limitation was that this study was not a randomized trial. However, there were no significant differences in baseline characteristics between the two groups, and multiple regression analysis was performed by making eGFR the dependent variable to estimate the effect of the switch from allopurinol to febuxostat on eGFR. The second limitation was not having evaluated urinary protein because there were missing values. However, the difference between the serum albumin levels of the two groups was not significant, and the multiple regression analysis included serum albumin.

## Conclusion

The switch from allopurinol to febuxostat reduced their serum UA levels and slowed the progression of renal disease more than in the group which allopurinol was continued in the cohort of hyperuricemia patients with advanced CKD who were treated with allopurinol. These results warrant future trials of XO inhibitors including allopurinol and febuxostat in hyperuricemic patients with advanced CKD.
